# Opening the research agenda for selection of hot spots for human biomonitoring research in Belgium: a participatory research project

**DOI:** 10.1186/1476-069X-9-33

**Published:** 2010-07-06

**Authors:** Hans Keune, Bert Morrens, Kim Croes, Ann Colles, Gudrun Koppen, Johan Springael, Ilse Loots, Karen Van Campenhout, Hana Chovanova, Greet Schoeters, Vera Nelen, Willy Baeyens, Nik Van Larebeke

**Affiliations:** 1 Faculty of Political and Social Sciences, University of Antwerp Belgium; 2 Vrije Universiteit Brussel, Brussels, Belgium; 3 Environmental Toxicology, Flemish Institute of Technological Research, Mol-Belgium; 4 Faculty of Applied Economics, University of Antwerp, Belgium; 5 Environment and Health, Flemish Government, Environment, Nature and Energy Department, Brussels, Belgium; 6 Flemish Agency for Care and Health, Division of Public Health Surveillance, Brussels, Belgium; 7 Provincial Institute of Hygiene, Antwerp, Belgium; 8 Study Centre for Carcinogenesis and Primary Prevention of Cancer, Department of Radiotherapy, Nuclear Medicine and Experimental Oncology, University of Ghent, Belgium

## Abstract

**Background:**

In order to select priority hotspots for environment and health research in Flanders (Belgium), an open procedure was organized. Environment and health hotspots are strong polluting point sources with possible health effects for residents living in the vicinity of the hot spot. The selection procedure was part of the work of the Flemish Centre of Expertise for Environment and Health, which investigates the relation between environmental pollution and human health. The project is funded and steered by the Flemish government.

**Methods:**

The involvement of other actors than merely experts is inspired by the 'analytical-deliberative' approach of the National Research Council in the United States and the extended peer community approach. These approaches stress the importance of involving different expert- and social perspectives in order to increase the knowledge base on complex issues. In the procedure used in the project a combination of expert and stakeholder input was essential. The final decision was supported by a multi-criteria analysis of expert assessment and stakeholder advice.

**Results:**

The endeavour was challenging from the start because of the complicated ambition of including a diversity of actors, potential hotspots, concerns and assessment criteria, but nevertheless the procedure proved its value in both structuring and informing the decision-making process. Moreover the process gained the support of most actors participating in the process, even though the final selection could not satisfy all preferences.

**Conclusions:**

Opening the research agenda exemplifies the value of inter- and transdisciplinary cooperation as well as the need for a well-structured and negotiated procedure that combines relevant factors and actors with pragmatism. The value of such a process also needs to prove itself in practice after the procedure has been completed: the tension between an ambition of openness on the one hand and a more closed attitude amongst experts on the other will continue to play a role even after closure.

## Background

Opening the research agenda to outsiders is not common practice for scientists and policy makers, especially in a complex and socially important field such as environment and health [1]. Science, and even policy oriented science, is commonly considered to be 'an expert affair' [2]. In this paper we present one of the exceptions. Within the context of environment and health research in Flanders, this ambition of opening up was formulated with respect to the selection of priority hotspots. Environment and health hotspots are strong polluting point sources with possible health effects for residents living in the vicinity of the hot spot. The ambition not only crosses boundaries of the research sphere of scientists, but also enters the domain of policy making, as in this case the research is carried out in close cooperation with representatives from policy making bodies. The endeavour thus entails a combination of both scientific and policy relevance as well as openness with respect to the input of 'third parties'. As is often the case, such idealistic ambition is relatively easily put down on paper when applying for research funding, but proves to be quite a challenge in practice, where even the ambition itself will become the subject of intense debate, even after the closure of the procedure.

### Human biomonitoring and the Centre of Expertise for Environment and Health

The Flemish Centre of Expertise for Environment and Health (CEH) [3] investigates the relation between environmental pollution and human health. The project is funded and steered by the Flemish government. The CEH brings together environmental health experts from all Flemish universities and two research institutes cooperate together with a social scientific expert unit which focuses on risk communication, risk perception, and on process aspects of knowledge production, interpretation, deliberation and cooperation between different scientific disciplines and other social actors.

The main research activity of the CEH is human biomonitoring [4-6]: measuring certain selected pollutants and their possible related health effects in human beings, through the use of biomarkers. A biomarker is a distinctive biological or biologically derived indicator (such as a biochemical metabolite in the body) which participates in some metabolic pathways and can disrupt them because its concentration is too high (e.g. copper is a required component of many redox enzymes but becomes toxic at high concentration) or because it substitutes the natural compound (e.g. dioxins also react with the AhR receptor, hence acting as an endocrine disruptor). The current biomonitoring program builds further on the previous campaign (2001-2006) and aims to follow internal pollutant levels trends in the Flemish population. It consists of two main pillars. First, reference values (population levels) for the Flemish population will be obtained in a representative population sample for a broad series of pollutants. Second, targeted human biomonitoring will be performed in specific hot spots: strong polluting point sources where the health status of residents living in the vicinity of the hot spot will be studied. In this paper we focus on the selection of hotspots.

## Methods

We will discuss here the main ambitions of the hotspot selection procedure, and how these were conceptualized. Because the development of the procedure (and the method) is also part of the research practice, we will limit ourselves here to sketching the ambitions and conceptual design laid down in the research plan of the CEH [7]. In the results section we will focus on the practical evolution of the ambitions, the procedure and the outcomes in research.

### Ambitions

Reference values (population levels) for the general population may be interesting for policy makers in order to enable them to follow time trends, to evaluate further needs or assess the efficacy of environmental measures. Defining and characterizing the exposure levels among high risk groups may be of even higher priority. High-risk groups may be subpopulations living in areas with elevated environmental exposure e.g. industrial areas, areas in the vicinity of waste sites, historically polluted areas [8], or they may be populations belonging to specific social classes [9], with specific dietary habits e.g. elevated fish consumers [10] or with a specific home environment [11].

High-risk subjects may be a high interest group for surveillance, as they are the most likely target to benefit from policy changes. Also, high-risk groups may be the first to show any effects from an intervention, long before it is reflected in the general population, thus acting as indicators of the effects of policy measures taken as to lower the health effects of environmental pollution. Another advantage of including high risk groups is that they are likely to provide the most informative links with exposure. As a consequence it may be easier to link biomonitoring (of high-risk subpopulations) with environmental and health data. They are likely to signal sources of high exposure or behaviours that lead to high exposure and disease events.

Many specific cases can be envisaged e.g. areas with high population density, areas around industries, areas with documented high environmental loads, areas of previous concern following an earlier biomonitoring study, areas with perceived health concerns. Due to time and budget constraints however choices have to be made: not all problematic cases can be investigated considering limited resources. To make these choices a procedure is to be developed based on two starting points:

1) Transparency of the selection procedure to stakeholders and

2) Participation of stakeholders in the selection process, and consultation on their viewpoints and arguments.

The involvement of other actors than merely experts is inspired by the 'analytical-deliberative' approach of the National Research Council in the United States [12] and the extended peer community approach [13]. These approaches stress the importance of involving different expert and social perspectives in order to increase the knowledge base on complex issues. The question is, however, how these different (technical) expertises, public preferences and values should be combined and judged. "Who can claim the right to select the expertises and values that should guide collective decision-making, in particular when the health and lives of humans are at stake?" [14] In general three main goals for involving public participation in decision-making processes and policy-relevant research may be considered [15,16]. First, the value of a final decision is higher when non-scientific (e.g. local) expert knowledge is included, since science itself suffers from many uncertainties and unknowns, especially with respect to complex issues such as the complex relationship between environment and health. Second, the legitimacy of the final outcome is higher when potentially affected parties can state their own case before their peers and have an equal chance to influence the outcome. Participation is therefore likely to increase public support for the policy decision-making process. Third, it is a way of implementing democracy.

As well as contributing knowledge, stakeholder involvement may also make a valuable contribution to the interpretation of knowledge or problem information, especially with respect to complex issues [17]. How do stakeholders navigate in the midst of complexity and uncertainty? Different social perspectives on what at first sight may be seen as unambiguous 'knowledge', 'problems', or 'data', may lead to totally different interpretations. Ney [17] does not believe that merely putting different stakes at the negotiation table will unambiguously solve problems when problem framing and the definition of problem solving strategies are characterized by differences of opinion. Nevertheless he perceives diversity of viewpoints to be an important source of relevant information for policy learning: conflicting interpretations act as vehicles for translating science to policy. It not only helps policy makers in processing the huge amount of information that normally comes with complex issues, but also helps focus on the issues from a different, non scientific, social, and more policy-relevant perspective.

Openness towards third parties in the selection procedure can thus support choices made within the environmental health surveillance. In principle, 'health relevance' should guide prioritisation, but direct health impact of environmental loads (well known health effects - despite combined exposure conditions) is difficult to demonstrate unequivocally. For some pollutants such as lead and cadmium health based reference values do exist, and exceeding these is generally agreed to pose an increased health risk. In most cases selection of 'monitoring cases or areas' will be based on societal arguments related to documented environmental data and perceived health concerns. Experiences from the 2001-2006 biomonitoring program clearly demonstrated the strategic value of choices of 'biomarkers' and 'regions'. Prerogatives need to be identified, explained and argued. The CEH also recognises the opportunities of a selection procedure for an increasing awareness of environmental health issues and enhanced trust of involved partners and the public in general as a positive impact. Instead of inviting the society at large to give its opinion on environment and health in general, involvement in a selection procedure will have practical relevance and involve specific cooperation with several actors.

### Procedure

With respect to policy interpretation of the 2001-2006 biomonitoring research results an action plan was worked out [18]. The hot spot selection procedure (for an overview see Table 1) should identify and argue the choices, made for specific surveillance activities and linked scientific research. The philosophy behind the selection procedure, described in this article, however, is similar to that of the action plan: in addition to the expert opinion, a round of stakeholder consultation and deliberation complements the decision making. Examples of relevant stakeholders are: environmental and health experts, trade unions, companies and their organisations, environmental groups, local residents' organisations. Examples of assessment criteria are: the size of the potentially exposed target groups, the severity of the potential health effect, existing environmental data which indicate potential exposure risks, information from previous biomonitoring campaigns, the vulnerability of target groups such as children or socially vulnerable inhabitants in polluted areas and reflections on environmental justice, scientific feasibility of the research, costs, ethical considerations, policy needs for follow up of environmental measures, policy perspectives for taking action, political or public controversy.

**Table 1:** Overview selection procedure

Step	Topic	Who is involved?
1	Call for candidate hotspot cases	Wide diversity of Flemish actors

2	Pre-selection of cases	CEH

3	Desk research	CEH

4	Expert elicitation	CEH and external experts

5	Expert synthesis	CEH

6	Stakeholder jury	Flemish national stakeholder organizations

7	Final decision: priority ranking of hotspot cases	CEH

8	Evaluation	CEH and all involved in the procedure

The basic problem that needs to be solved is choosing between options for hotspot research that are rather different in nature. The choice is being based on incommensurable assessment criteria: criteria with respect to public health aspects, policy aspects, social aspects and research aspects. By incommensurability we mean that these aspects do not share likewise measures that make comparison easy. An obvious structuring method for this is a multi-criteria method of analysis [19,20]. The assessment procedure is organised as follows: first, desk research provides the different options with background information concerning the different assessment criteria. The environmental and health information relevant to assess the health risk and research aspects is currently being gathered by the natural scientists. The social scientists are responsible for policy aspects and social aspects. Second, based on desk research information, experts with regard to environment and health assess the health risk criteria, policy experts the policy aspects and social experts the social aspects. These assessments result in both quantitative information (priority rankings of options on different criteria) and qualitative information (arguments, difference of opinion, uncertainties). The outcomes of the expert consultation are processed in a multi-criteria analysis as well as in an account of (other) qualifications. Third the results of both desk research and expert consultation are discussed by a stakeholder jury that gives advice on the basis of all information (whereas experts only assess issues that more or less belong to their particular field). The procedure is aimed at ensuring a well informed and substantiated decision-making process by the CEH.

## Results

We will describe here how the hotspot selection procedure developed in practice: from translating the concept into practice to the definitive start of the research. We will notice that some of the important selective decisions were even taken after the selection procedure had been completed.

### Definition of hot spots and actors involved in the procedure

In cooperation with both scientists and policymakers involved in the CEH it was decided, after intense debate, that in order to qualify as a hotspot a proposed case, irrespective of who proposed it, must fulfil three criteria:

1. There must be evidence, or at least an indication, of an environmental impact on public health.

2. Human biomonitoring, enabling levels of pollutants in humans and their health effects to be measured, must be a scientifically appropriate tool for addressing the problem.

3. There is a perceived need for policy-oriented research with a view to possible remedial action.

With respect to actors to be invited to propose candidate cases for the procedure, the decision was made to be open to a broad range of people and institutions: scientists, policymakers, stakeholders and the general public. The strategy for reaching as many potential actors as possible was via the 'snowball' method, i.e. through intermediate contacts by which e.g. local medical environmental experts who we work with were asked to spread the call for proposals amongst whoever they considered to be interested. Among the organizations that were contacted in order to try to be as open as practically possible to a diversity of actors also were environmental groups, a network of poor peoples' organizations and a network of initiatives addressing vulnerable groups. The latter two organizations specifically were included as to open the possibility of marginalized populations and vulnerable actors to be involved in the stage of hotspot proposals. All contacts were specifically asked to spread the call for proposals further to whomever they thought might also potentially be interested.

### Response

Towards the end of 2007, a call for the submission of cases by scientists, policymakers, administrations and civil society organisations yielded a list of 85 very diverse potential hotspots. Some of these cases concerned either a health issue that was possibly or presumably (co)determined by an environmental factor or an environmental issue causing health concerns among local residents. Among the proposed focal points were specific pollutants or industrial plants, industrial zones, fine particulate matter, traffic, dumping sites, pollutants in home-grown vegetables, noise pollution, the risks posed by drinking water contamination, and the effects of pesticides and herbicides. The cases were submitted by 35 organisations. The majority consisted of representatives of public bodies (government and local level) and local medical environmental experts (sometimes in conjunction with local authorities). The second largest group of respondents (about 20%) consisted of scientists external to the CEH. Only two respondents with other social backgrounds participated: an environmental interest group and a patient group.

### Pre-selection of cases

From the 85 proposed cases, the CEH selected a shortlist of nine cases on the basis of research-related criteria and additional information obtained from various government agencies. The research related criteria used at this stage were the following:

- Is the problem researchable by means of human biomonitoring?

- Does the hotspot concern a clearly demarcated area?

- Is it to be expected that lessons can be learned from the hotspot case on a more general level than just local level?

- Can we group different cases under the umbrella of one type of hotspot?

- Does the pre-selection sufficiently reflect the diversity of submitted case proposals?

- Do cases already have sufficient scientific attention in other (current or recent) studies?

The nine cases that were pre selected for the analytical deliberative procedure:

- Mortality - Dendermonde: The governmental health indicators show that since 1990 the district of Dendermonde has had a higher standardised mortality ratio than other Flemish districts. This observation has caused concern among local residents.

- Brominated compounds - Oudenaarde: Measurements have shown increased levels of brominated flame retardants in freshwater fish in the Upper Scheldt at Oudenaarde. Concentrations measured in this location in 2001 were among the highest in the world. The question arises whether levels of brominated flame retardants in humans are also elevated in this region.

- Dumping sites: Dumping sites contain a complex cocktail of chemical agents, with potential health effects in local residents, and consequently cause considerable public concern.

- Industrial zone Ghent: Industry and related activity in the Ghent Canal Zone cause pollution that is potentially harmful to local residents. Concern has centred mostly on emissions of fine particulate matter. Environmental measurements have thus far not yielded unequivocal answers to questions regarding local health impacts.

- Industrial zone Antwerp harbour: The port of Antwerp is the world's second-largest petrochemical cluster, after Houston, Texas. Moreover, these petrochemical plants are located in the immediate vicinity of residential areas.

- Industrial Zone Genk: The Genk Zuid industrial zone is characterised by a mixture of industrial activity and emissions: a stainless steel plant, a car assembly plant and its suppliers, a glue production plant, a chipboard plant, and a coal and biomass-powered electricity facility. Concentrations of nickel and chrome in fine particulate matter have been found to be particularly high in comparison with levels measured in other Flemish locations. The industrial estate is entirely surrounded by a residential area. A recent health survey has shown that local residents are concerned about the health impact of industrial pollutants.

- Benzene Geel: A local factory produces tonnes of benzene as a by-product of paraxylene production. Benzene is a known carcinogenic, giving cause for concern amongst local residents.

- Shredder Menen: The scrap-processing industry of Menen is an international player in this specialised market. Measurements in deposited air particles, soil, vegetables and cows milk show elevated concentrations of dioxin-like PCBs and dioxins. Emissions of metal-containing particulate matter are also substantial.

- Chipboard plants Western Flanders: There are a number of chipboard plants in West Flanders Province, emitting dioxins, fine particulate matter and possibly also solvents and formaldehyde.

These cases were further assessed in the selection procedure in which a multi-criteria analysis was used for structuring all relevant information.

### Hot spot selection analysis: desk research

We conducted desk research with respect to each of the shortlisted cases, focussing on four main aspects: public health aspects, social aspects, policy aspects and research aspects. The public health aspects were investigated by the natural scientific experts of the CEH and were mainly based on available data in Belgium and international literature. For the social aspects desk research material was less straightforward. We decided to collect information from local environmental health workers as they can provide valuable insight into the societal dimension of cases relevant to their working region and involving local actors. The local environmental health workers are part of a broader environment and health network of which as well as the CEH central government is also part. We sent them (n = 12; 11 responded) a questionnaire asking to make an assessment of:

- Local problem perception:

○ Do local actors know about the problem?

○ Do they complain about the problem?

○ Are they concerned about the problem?

- Local support for:

○ Scientific environment and health research in the hotspot area

○ Human biomonitoring in the hotspot area

○ Policy action in the hotspot area

The list of local actors to be involved in our desk research was made up of an inventory of relevant local actors per hotspot case (e.g. local residents groups, general practitioner associations, industry, environmental groups and local authorities) supplied to us by the local environmental health workers and our own inventory. We invited local actors (n = 296) asking questions on:

- How do they assess the problem?

- Which information do they have about the problem?

- How severe is the potential public health risk?

- How should the problem be dealt with?

○ Research or remedial action?

○ Pros and cons with respect to human biomonitoring in the hotspot area

**Figure 1:**
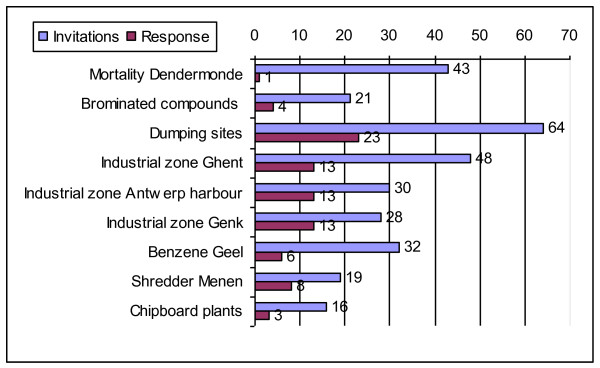
**Invitations and response to second questionnaire local actors**.

The response rate for the second questionnaire differed a lot per hotspot region: see Figure 1. A total of 84 local actors responded of which most were located near dumping sites, albeit in different locations throughout Flanders.

**Figure 2:**
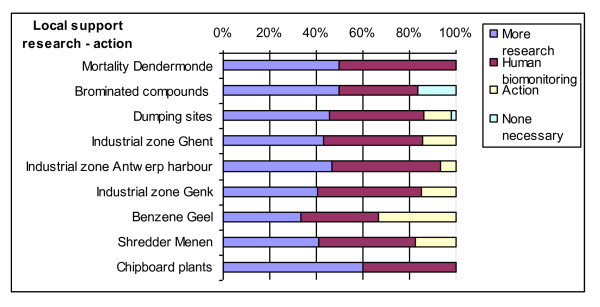
**Local support action - research**.

We will not present all results of both questionnaires here, but limit ourselves to the balance research - action (Figure 2) and to the pros and cons with respect to the human biomonitoring voiced by the local actors (Table 2).

**Table 2:** Arguments pro and contra human biomonitoring

Arguments pro human biomonitoring:	Arguments contra human biomonitoring:
A need for scientific knowledge	The problem is well known
	
	The problem is already under control
	
	Practical constraints: money and time consuming and privacy sensitive
	
	Technical constraints: requires a big group and sensitivity of measurement is problematic
	
	Results may be difficult to interpret

It can have a catalytic effect for problem solving action	Research should not slow down problem solving action

It addresses the worries of neighbouring people	It may cause panic and unnecessary concerns

An investment in a better image for the region	It can give the region a bad image

Awareness raising effect	
	
The problem is serious	
	
It evaluates effect of (ongoing) policy actions	
	
There is local support for it	

We can clearly see (Figure 2) that most responding local actors are in favour of more research (of which most are also in favour of human biomonitoring), even though some bring forward strong arguments against human biomonitoring (table 2), including the fact that in some cases the time for action is now as enough is already known about the problem. On the other hand even in cases that have a longer history with lots of research and media and policy attention, human biomonitoring is sometimes expected to supply new knowledge (public health effects) as well as offering the opportunity for renewed attention leading to a possible catalytic effect.

Regarding policy aspects similar to a previous procedural exercise (on interpretation of research results) [18] we decided that policy issues are too complex and too extensive to be mapped in desk research considering the practical constraints such as limited time and man power. We therefore decided to approach these issues via a questionnaire focussing on relevant aspects of environment and health policymaking and to address these to experts with field experience. With respect to the research aspects in this procedure we took a similar approach in posing this question also to the experts, be it the environment and health scientists we were to consult regarding the public health aspects.

### Expert elicitation

On the basis of the desk research findings, the nine hotspots were assessed by three types of experts: environmental and health experts (environmental and health scientists; n = 5), policy experts (experts working for governmental bodies or governmental expert institutes; n = 7) and social experts (social scientists; n = 7). As with a similar assessment procedure performed previously within the framework of the CEH [18] for the selection and recruitment of experts three main principles were of importance:

- Diversity: because of the complexity of the issue, a diversity of expertise is desirable

- Openness: to guarantee the independence of the expert advice, the threshold for participation has to be low

- Practical feasibility: the process must be manageable in consideration of resources such as time, money, manpower

After intense debate, however, we made one exception: our natural scientific colleagues in the CEH pushed to assess the Public health and Research aspects themselves. Their main arguments were that within the CEH there was sufficient expertise on these issues and this would make the practical application of the procedure easier. We see here a major move away from the appreciation of independent external reflection from outside experts. We will come back to this phenomenon of traditional expert reflex in the discussion section.

The experts were asked, each in respect of their field of expertise only, to assess the proposed cases in relation to the following three groups of criteria (Table 3):

**Table 3:** Three groups of criteria

Public health and research* aspects	Policy aspects	Social aspects
Health effects	Policy relevance of this type of knowledge	Public concern

Importance of the study for public health in general	Feasibility policy action at the source	Public support

Feasibility of the research*	Feasibility prevention or treatment health effects	Problem solving: research or action?

	Arguments pro and con human bionmonitoring	Arguments pro and con human bionmonitoring
	
*The research aspects were integrated in the Public health assessment: feasibility of the research.	Short term feasibility policy action	
		
	Relation with current policy	

Based on information from the desk research (with the exception of most policy aspects and research aspects, where the process involved mainly asking questions) the experts were asked to assess the cases with respect to the different sub criteria on a qualitative response scale of 7 items, resulting in rankings of hotspot cases. For example regarding the importance of human biomonitoring: Very important - Important - Fairly important - Fairly unimportant - Unimportant - Very unimportant - *Do not know*

We also asked them to explain their arguments and any type of assessment uncertainty. Furthermore the experts were asked to give weights to the (relative importance of) respective sub criteria. In a multi-criteria analysis the combination of individual expert rankings and weightings resulted in overall expert group consensus rankings regarding the priority of hotspot cases (Table 4). The overview of consensus rankings presents us a rather mixed picture. No hotspot case clearly scored high on all criteria. Several score high on one or two criteria but considerably lower on other: e.g. Mortality Dendermonde and Shredder Menen. Only one case scores consistently (rather low) on all criteria: Chipboard plants. Based on this picture, thus we cannot draw clear conclusions for an unambiguous ranking of hotspot cases. Moreover we have to take into account the fact that not all criteria will be considered of equal importance by different actors.

**Table 4:** Overall expert group consensus rankings priority hotspot cases

*Criteria Hotspots*	Health*	Policy	Social	Research feasibility
**Mortality Dendermonde**	1	7	4	5

**Brominated compounds**	3	1	5	5

**Dumping sites**	2	2	5	6

**Industrial zone Ghent**	2	5	2	1

**Industrial zone Antwerp harbour**	5	5	1	3

**Industrial zone Genk**	3	4	3	2

**Benzene Geel**	3	3	1	1

**Shredder Menen**	5	6	1	4

**Chipboard plants**	4	4	6	5

*Without taking into account the sub criterion of research feasibility

Finally we also asked experts on their views of the pros and cons with respect to human biomonitoring that were highlighted in the questionnaire for local actors (Table 2). We presented the pros and cons local actors pointed out for the different hotspot cases and asked the experts which elements they considered the most important regarding the selection of hotspot cases. We asked this because we were curious how experts would respond to lay input, and as such experiment with co-consideration. The experts clearly pointed out two pros as being most relevant: 'the need for more problem knowledge' and 'the seriousness of health risks'. Regarding cons the clearest result is on issues not considered to be very relevant by the experts: 'practical constraints', 'fear of panic' and 'the risk of a bad image for the region'.

We made an effort to translate the pros and cons with respect to human biomonitoring considered as most important to rankings of hotspot cases, in order to facilitate integration of these aspects into the overall assessment of cases. This was not at all straightforward as these pros and cons with respect to human biomonitoring did not necessarily have the same (pro or con) meaning when relating to different cases. Moreover it seemed that different types of experts had different, sometimes even opposing interpretations regarding similar pros and cons with respect to human biomonitoring, the clearest example being the difference between environment and health experts on the one hand and policy experts on the other. This was especially noticeable regarding 'the need for more knowledge on the problem'. Whereas environment and health experts mainly interpreted 'the need for more knowledge' about the problem as a challenge, policy experts interpreted this more pragmatically with respect to policy relevance. For example regarding the case 'Mortality - Dendermonde', the lack of knowledge about the cause of a higher standardised mortality ratio for the environment and health experts is a reason to investigate the hotspot. For policy experts though, the lack of a clear indication that there is a relation with environmental causes is a reason not to investigate the hotspot by means of environment and health research. Another example regarding the Chipboard plants case shows a high ranking for policy experts because of clear health effects, whereas environment and health experts seem less interested precisely because of the clarity of the health effects. These two types of experts clearly focus on different targets: problem knowledge - problem solving.

### Expert synthesis

Based on procedural experience with stakeholder consultation on the interpretation of research results [18] we decided to try a different approach; one that was requested by the stakeholders involved in that exercise. Instead of presenting the stakeholders with all the information gathered in both desk research and expert elicitation and ask them to decide what to do with it, they proposed an intermediate step in which the organizers of the procedure (the experts at the CEH) would put together a more concrete proposal with argumentation on which the stakeholders could reflect more pragmatically in a group discussion. For designing this expert synthesis we gathered all the information in as structured and condensed a way as possible so as to both fully inform and structure discussion amongst the CEH experts. We presented a document in which the following information was presented in table format:

- Rankings of cases based on all criteria:

○ A ranking with equal weight for all criteria

○ Rankings with different weights for all criteria

○ Rankings based on sub criteria

- Provisional rankings of cases based on expert preferences with respect to main arguments pro or con human biomonitoring

- An overview of all arguments expressed by the experts

- An overview of types of assessment uncertainty expressed by the experts

During the discussion the choice of most important cases was based mainly on the results from the expert elicitation. Especially the rankings and arguments regarding the public health aspects carried weight in the discussion. Also the need for knowledge for policy was considered important in this respect. Furthermore the aspects of local public support, familiarity of the problem and practical research feasibility especially were also addressed in the discussion. We must clarify that although this partly concerned the same experts as were involved in the earlier expert elicitation (on public health and research aspects), the bigger group discussing the overall synthesis also involved other experts who had not taken part in the earlier expert elicitation. Table 5 shows the ranking that resulted from the discussion with some of the main arguments. The fact that the arguments presented in the overview often seem similar but leading to different ranking positions can be explained by more detailed argumentation and that comparisons between hotspot cases are made. The relatively low ranking of the industrial zone of Ghent e.g. also has to be explained by some research concerns that the actual research in this case would be rather complex in comparison to e.g. the industrial zone of Genk case or the Shredder Menen case. This illustrates that the selection process based on further discussion and new insights as well as reflection based on previous steps in the procedure shows its iterative nature. Moreover does it show that it is often not very easy to discriminate between different environment and health hotspot cases.

**Table 5:** Main arguments hotspot ranking

*Ranking Hotspots*	Main arguments
**1. Industrial zone Genk**	Severity of public health risks, need for knowledge for policy and local public support

**2. Shredder Menen**	Need for knowledge for policy and local public support

**3. Mortality Dendermonde**	Severity of public health effects
	Need for knowledge about the cause

**4. Chipboard plants**	Need for knowledge for policy

**5. Industrial zone Ghent**	Severity of public health risks and local public support
	Complexity of research in this case

### Stakeholder jury

The composition of the stakeholder jury was inspired by advisory bodies in Flanders such as the Flanders Social and Economic Council and the Flanders Advisory Council on Environment and Nature. In addition organisations with a focus on the health perspective were of interest for us as well as consumer organisations because of the relevance of a consumer perspective. We invited the following 15 stakeholder organisations by email: three employer organisations, two agricultural organisations, three labour unions, two environmental organisations, one platform of patient groups, one association of general practitioners, two consumer organisations and the Flemish network of local health and environmental experts. We want to point out that local residents were not invited as stakeholders for this stage of hotspot selection. From a community-based participatory research perspective [21] as well as from a environmental justice perspective [22] this can be questioned, as one of the reviewers of this paper rightfully did. With respect to the latter it would indeed have been an opportunity to also have invited the network of poor peoples' organizations and the network of initiatives addressing vulnerable groups that were amongst the big groups of organizations and contacts that were consulted in the first stage of the process when the candidate hotspots were collected. It was decided though to only involve Flemish organizations with some connection to the fields of environment and health. With respect to local residents it was decided that as the decisions concerned comparison of candidate hotspots on a national level, and not the local level, it would not be relevant to invite local actors with respect to all candidate hotspots involved at this stage of the procedure. Moreover this would not be honest with respect to cases in which e.g. local residents might be less well organized, e.g. because of the novelty of environment and health issues in their local setting. It is the intention though of the CEH to have special attention for the involvement of local residents and more vulnerable groups in the actual hotspot research in the cases that eventually will be selected on the basis of the selection procedure discussed in this paper.

Representatives of eight organisations participated: two employer organisations, three labour unions, one environmental organisation, one platform of patient groups, one association of general practitioners and one consumer organisation. The jury discusses the outcomes of the expert synthesis described in the previous section. After intense discussions, in which also extra background information was requested and presented all participants (some afterwards as they had to leave earlier) made up a list of top three priority cases: Table 6.

**Table 6:** Jury members' individual hot spot rankings

*Jury member's individual rankings Hotspots*	A	B	C	D	E	F	G	H
**Mortality Dendermonde**					3	3	2	

**Brominated compounds**				2				

**Dumping sites**				3				

**Industrial zone Ghent**	2	2			2			1

**Industrial zone Antwerp harbour**								2

**Industrial zone Genk**	1	1	1		1	1		3

**Benzene Geel**								

**Shredder Menen**		3	2			2		

**Chipboard plants**								

**New: traffic**	3		3	1			1	

Clearly views differed substantially, except for the case of 'Industrial zone Genk' which got broad support and the cases of 'Benzene Geel' and 'Chipboard plants' clearly got no support at all. Compared to the expert synthesis and the broader list of cases reviewed in desk research and expert elicitation a new case was proposed by several jury members: traffic. This (type of) case was also amongst the original list of cases proposed by a wide diversity of actors at the beginning of the procedure, but excluded by the experts of the CEH because human biomonitoring in such a case is considered to be rather complicated technically. This was also a reflection of one of the CEH-experts attending the jury meeting. Nevertheless half of the jury members selected it in their top three.

### Final priorities: on paper and in practice

In Table 7 we present the final outcomes of major decisions: the ranking based on the expert elicitation, the ranking based on including the jury input and the final outcomes in research practice. We notice quite a few substantial changes: what happened along the way?

**Table 7:** Final priorities: on paper and in practice

A. Ranking based on expert elicitation	B. Ranking based on expert round and jury	C. Order in research practice
1. Industrial zone Genk	1. Industrial zone Genk	1. Industrial zone Genk

2. Shredder Menen	2. Mortality Dendermonde*	2. Shredder Menen

3. Mortality Dendermonde	3. New: traffic*	3. Industrial zone Ghent

4. Chipboard plants	4. Shredder Menen	
	
5. Industrial zone Ghent	5. Industrial zone Ghent	

*Because the experts of the CEH were not completely sure about the relevance or feasibility of human biomonitoring in these cases, it was decided to await results of a pre-investigation looking into this before deciding to actually implement this ranking in research practice.

Step A to B clearly shows the influence of the advice of the stakeholder jury: the 'Mortality Dendermonde' case gained priority over the 'Shredder Menen' case, which was also surpassed by the new case 'Traffic' that was proposed by several jury members. However, step B to C shows the disappearance of both cases in actual research practice. After further investigation for both cases it was decided that human biomonitoring is currently not a good option. For the 'Mortality Dendermonde' case the main reasons were that there were no indications found for (recent) environmental problems and that clues were found for life style causes probably related to the high standardised mortality ratio, which has no direct relationship with environmental problems. For the 'Traffic' case it was concluded that the current technical means for human biomonitoring for such case are not mature enough and there is need for further development in this area. In the end the government responded to this call by promising future funding for this technical development so as to make such a hotspot investigation potentially more worthwhile. Currently the top two hot spot cases are up and running. Whether a third case is possible in the current timeframe of the CEH (funded until 2011) is uncertain at this moment.

### Evaluation

Part of our way of working is inviting everyone involved in the project to an evaluation the project. We will briefly address how different actors assessed the project. The large majority of actors involved at the stage of proposing the initial candidate case at the beginning of the project are very positive about the project and about the openness towards different social groups. This involvement creates opportunities for a more integrated approach and for the inclusion of specific local knowledge and a feel for local concerns. Involvement moreover is considered to be functional in creating support for the research.

The large majority of local actors involved in the desk research phase with respect to social aspects are also very positive about the project as well as the involvement of social groups. Some though express the criticism that their involvement at that stage is too late in the process; they want to be involved from the start. An interesting difference of opinion that was apparent in this evaluation is that one respondent warns us for not equating perceptions with seriousness of health risks, whereas another stresses the importance of involving social factors. Benefits of the approach that are mentioned focus on the value of local information/knowledge, the right to access to information for local actors, generating local support and awareness-raising. Critical remarks that are highlighted concern the question as to whether some form of representation can be guaranteed with respect to involvement, worries about the objectivity of input, the emotional side of some contributions, the question as to whether local involvement will indeed lead to new and valuable issues being raised and the question as to whether such involvement should not be postponed to a later stage, when research has been done and policy interpretation is at stake.

In addition, the experts involved in the expert elicitation were asked evaluative questions. Regarding the involvement of social groups the environment and health experts are positive; some call this an innovative approach. The policy experts are also positive in this respect. Some remarks are made on the need for an independent involvement of stakeholders and one respondent is a bit hesitant with respect to emotional issues related to such involvement. The majority of social experts are also positive on both procedure and involvement. One is critical in two respects. First the quality of the information basis from the desk research is questioned because of the low number of respondents taking part in the questionnaire of local actors. Second the separation between expert assessment and jury assessment is criticized: why not integrate both judgements?

The stakeholders taking part in the jury generally are positive about the procedure. The involvement of local actors is considered to be important. Also their own involvement is appreciated as the issues dealt with had important social relevance. Moreover it is considered a good way of informing and involving them with respect to the research.

## Discussion

When we look at the outcomes of the selection procedure, from an outsider perspective one may cynically draw the conclusion that in the end the opening up of the research agenda merely came down to window dressing or from a cynical expert perspective it came down to causing a lot of bother for nothing - so bother? The cases that are finally being worked out in practice can be qualified as rather traditional hotspot cases: traditional in the sense of aiming at what based on current scientific knowledge and technology is relatively easy to measure and interpret according to traditional scientific standards of problem information and statistics. A messier but apparently socially (equally) important case such as traffic is discarded because of being much more difficult to investigate. Researchers do have legitimate grounds for this of course; they have chosen to invest in the medium term quality of strengthening research capabilities with respect to the traffic hotspot in order to better be able to make a difference in complicated traffic debate in the longer run. Others may see this though as a classic example of a traditional expert reflex aiming at what is technically and methodologically feasible from the perspective of traditional research standards, arguing that from a problem solving perspective in focussing on complex reality a different turn might have been taken. Internationally recognized experts such as Philippe Grandjean [23,24] and David Gee [25] challenge traditional reductionist approaches as well as the yardstick of perfect information and strict statistical standards with respect to environment and health research. Moreover, even in more traditional hotspot cases foreseen and unforeseen complexities will occur, challenging choices based on arguments with respect to research attainability and effectiveness. Therefore the question of how and if the challenge posed by the stakeholder jury of investigating traffic could have worked out in practice remains unanswered. This reflection does not mean that researchers were not brave enough to take up this challenge and that we can predict which solutions to this challenge might have been found or not found. We merely reflect on the fact that in this case the challenge from 'outsiders' was not fully accepted by the experts. This can also partly be explained by the pressure from policy makers to produce scientific knowledge that can live up to main stream scientific standards. Nevertheless the challenge will be addressed partially by investing in future research capabilities in this respect.

Parallel to this development the expert mood in project meetings has been ambivalent from the start with respect to involving outsiders in 'their expertise'. It is not easy for natural scientists to adapt to such new practice: the open arms of good will at the level of ambition have to be reconciled with the traditionally closed mindset of a lot of disciplinary routines and training. The complexity of other domains, disciplines, approaches, in this case involving other social perspectives and as such social complexity, is often underestimated and in need of sufficient time and effort in order to integrate and fine-tune them. This not only requires integration of ambitions and mindsets, but also of practices of very busy, sometimes overloaded, people, who all have to perform well according to the standards of their own working and peer-surroundings.

## Conclusions

We may conclude that what Kunneman [26] calls a 'place of effort' when dealing with complexity is an underestimated hotspot in itself: the complexity of processes like the one discussed in this paper are often underestimated in complexity. This not only concerns the effort needed as such, but also the intensity of differences of opinion and views amongst those involved and the sometimes heated debates that follow from this. Here the complexity is not only evident in the ambition of opening up the research agenda, but also in dealing with complex reality in research. In the case of natural scientific practice we may note that environment and health science to a large extent is firmly rooted in traditional scientific approaches. Individual experts are not to be blamed for this, they are trained in that tradition and they are judged and awarded in the spirit of that tradition, by peers, by journals, by those who fund their research. Nevertheless the limits of tradition with respect to solving complex real world problems are becoming more and more obvious [2], also in the field of environment and health research. Involving stakeholders from real world complexity is an important contribution to opening up traditional approaches to critical reflection and especially to a more problem solving orientation.

The effort of opening up the research agenda discussed here is a bold and relevant contribution, but in practice the effort of the CEH reveals difficulties on the road to ambition. Simultaneously step by step initiatives like the CEH will help grow the body of practical experience, by which a scientific approach really contributes to problem solving in complex environment and health issues. It represents a courageous attempt to take up this gauntlet, and so far there is no intention to put it down.

## Abbreviations

CEH: Centre of Expertise for Environment and Health

## Competing interests

The authors declare that they have no competing interests.

## Authors' contributions

All authors (except for JS who is not directly involved in the CEH) took part in general CEH discussions on this case study. More specific contributions and roles: HK coordinated the case study (procedure and research) and is author of this paper. BM collaborated in the case study (procedure and research); translated some raw material for the text. KC, AC and GK contributed to the pre selection and desk research; commented on the text. JS provided support on the multi-criteria analysis. IL proposed that the procedure developed for the action plan be used in this case study; commented on the text. KVC and HC are policy representatives in the CEH-steering group. GS is coordinator of the human biomonitoring work; commented on the text. VN coordinates the field workers of the human biomonitoring. WB is coordinator of the CEH and commented extensively on the discussion section. NVL is spokesman of the CEH and mainly participated as senior researcher in the pre-selection phase of the procedure. All authors approved the final version of the manuscript.
